# Ongoing Research on Microgreens: Nutritional Properties, Shelf-Life, Sustainable Production, Innovative Growing and Processing Approaches

**DOI:** 10.3390/foods9060826

**Published:** 2020-06-24

**Authors:** Massimiliano Renna, Vito Michele Paradiso

**Affiliations:** 1 Institute of Sciences of Food Production, CNR-National Research Council of Italy, Via Amendola 122/O, I-70126 Bari, Italy; massimiliano.renna@ispa.cnr.it; 2 Department of Biological and Environmental Sciences and Technologies, University of Salento, S.P. 6, Lecce-Monteroni, I-73100 Lecce, Italy

**Keywords:** bioaccessibility, bioactive componds, Brassicaceae, carotenoids, hydroponic cultivation, mineral elements, polyamine, quality, nitrate, wild edible species

## Abstract

Microgreens garner immense potential for improving the nutritional value of the human diet, considering their high content of healthy compounds. On the other hand, they are gaining more and more interest not only for their nutritional value but also for their interesting organoleptic traits and commercial potential. The purpose of this Special Issue is to publish high-quality research papers with the aim to cover the state-of-the-art, recent progress and perspectives related to production, post-harvest, characterization, and potential of microgreens. A broad range of aspects such as cultivation, post-harvest techniques and packaging, analytical methods, nutritional value, bioaccessibily and prospects are covered. All contributions are of significant relevance and could stimulate further research in this area.

“Microgreens” is a marketing term used to describe young and tender edible seedlings harvested when the cotyledonary leaves have fully developed and the first true leaves emerge. This category of vegetables presents different traits as compared to the already known sprouts and the common baby leaf vegetables [1,2].

Microgreens are gaining increasing interest as potential functional foods, due to their relevant contents of micronutrients and bioactive compounds [3,4,5,6,7]. They are gaining popularity also due to their varying and attractive colors, textures, and flavors [8]. The wide number of species and cultivars that can be grown as microgreens and the possibility to control their growing conditions even in micro-scale production underlie their promising potential for tailored nutrition [9], as well as to address particularly demanding categories of consumers, such as vegans or raw-foodists. At the same time, microgreens can be grown in a very simple way, even in very small spaces, being suitable for urban agriculture, as well as a component of space life support systems [10]. Nevertheless, several research themes still need to be explored, throughout the chain. A representative, though non-exhaustive, list of current research themes could include:

- sustainable cultivation and growing substrates from renewable sources;
- microgreens production in indoor, urban or space growing systems;
- nutritional characterization and effects of genotypes (i.e., biodiversity exploitation) and of growing conditions (e.g., nutrition, natural and artificial lighting systems, use of selected wavelengths);
- nutritional tailoring to address specific needs (e.g., nutritional integration for children, pregnant women, elderly people; chronic diseases management; prevention; hidden hunger issues);
- packaging and shelf-life, with specific focus on safety, nutritional content, environmental and sustainability issues;
- nutrient bioaccessibility and bioavailability;
- specific analytical methods for either characterization or quality control.

The purpose of this Special Issue was to collect contributes on some of these relevant themes and publish high-quality research papers with the aim to cover the state-of-the-art, recent progress and perspectives related to production, post-harvest, characterization, and potential of microgreens.

In the first article, entitled “Evaluation of the Bioaccessibility of Antioxidant Bioactive Compounds and Minerals of Four Genotypes of *Brassicaceae* Microgreens” by Beatriz de la Fuente, Gabriel López-García, Vicent Máñez, Amparo Alegría, Reyes Barberá and Antonio Cilla [11], the contents of minerals and antioxidant bioactive compounds were evaluated in broccoli (*Brassica oleracea* L. var. *italica* Plenck), green curly kale (*Brassica oleracea* var. *sabellica* L.), red mustard (*Brassica juncea* (L.) Czern.) and radish (*Raphanus sativus* L.) hydroponic microgreens. Authors evaluated the content of potassium, calcium, magnesium, iron, zinc, ascorbic acid, total soluble polyphenols, total carotenoids, total anthocyanins, total isothiocyanates and total antioxidant capacity. For the first time, the bioaccessibility of these compounds was also evaluated on microgreens by using a simulated gastrointestinal digestion process. The authors found that all four genotypes of microgreens provided relevant amounts of ascorbic acid and carotenoids, while mineral content was comparable to those reported in the literature for microgreens hydroponically grown. Moreover, the article reported that the greatest contributors to the antioxidant capacity after the simulated digestion were polyphenols and isothiocyanates, while macroelements showed high bioaccessibility values, reaching 90% in the case of calcium. Overall, the authors suggested that the four genotypes of Brassicaceae microgreens can be considered a good source of antioxidant bioactive compounds, although radish and mustard presented the highest bioaccessibility not only for these compounds but also for minerals.

The second contribution regards the “Antioxidant and Mineral Composition of Three Wild Leafy Species: A Comparison Between Microgreens and Baby Greens” by Anna Lenzi, Alessandro Orlandini, Roberta Bulgari, Antonio Ferrante and Piero Bruschi [2]. In this study, the authors compared three wild leafy species (*Sanguisorba minor* Scop., *Sinapis arvensis* L., and *Taraxacum officinale* Weber ex F. H. Wigg.), harvested at the microgreen and baby green stages, in order to evaluate yield and content of chlorophylls, carotenoids, anthocyanins, phenolic index, nitrate and mineral elements. The authors also calculated the potential contribution to human mineral intake, showing that both micro- and baby greens could positively contribute to the dietary intake of macro- and microelements as well as non-nutrient bioactive compounds, having a contribution comparable to, or even larger than that of vegetable crop species. On the other hand, the authors concluded that, although wild edible plants may play an important role in human nutrition, the observed high amounts of nitrate and traces of some metals potentially detrimental for health, suggest the need for caution in the use of wild species to produce microgreens and baby leaves.

The third paper illustrates the “Setup of an Extraction Method for the Analysis of Carotenoids in Microgreens” by Vito Michele Paradiso, Maria Castellino, Massimiliano Renna, Pietro Santamaria and Francesco Caponio [12]. In this research, a specific extraction procedure for the analysis of carotenoids in microgreens was developed, starting from the remark that the analysis of carotenoids is inherently difficult, and that extraction is the most critical step. Authors evaluated several aspects, such as the solvent composition, extraction time, solvent/sample ratio, and repeated extractions. The results enabled the authors to develop an effective protocol for the extraction and analysis of carotenoids from microgreens that allows the recovery of 97.2%, limits of quantitation of 5.2 μg g^−1^ for lutein and 15.9 μg g^−1^ for β-carotene, as well as intra-day mean repeatability of 5.7% and inter-day mean repeatability of 4.7%. The authors concluded that the developed protocol proved to be more efficient in the extraction of carotenoids from the delicate tissues of microgreens, even compared to another method from the literature. Therefore, the proposed analytical method could allow improvement in the obtainment of nutritional data on microgreens, which are claiming increasing attention for their functional potential and suitability for tailored nutrition.

The fourth article concerns the “Accumulation of Agmatine, Spermidine, and Spermine in Sprouts and Microgreens of Alfalfa, Fenugreek, Lentil, and Daikon Radish” by Irena Kralj Cigić, Sašo Rupnik, Tjaša Rijavec, Nataša Poklar Ulrih and Blaž Cigić [13]. This study was conducted firstly to determine the polyamine content in seeds, sprouts, and microgreens of three legumes (lentil, fenugreek, alfalfa) and daikon radish. The authors also evaluated whether microgreens are nutritionally superior to sprouts in terms of polyamine content. Furthermore, an evaluation of the enzymatic potential of sprouts to degrade undesirable biogenic amines was carried out. The authors observed that, in general, sprouting led to the accumulation of the total polyamine content. Alfalfa microgreens showed the highest levels of agmatine, fenugreek sprouts showed the highest content of putrescine and cadaverine, in lentil microgreens the highest content of spermidine was found, while fenugreek microgreens showed the highest content of spermine. Moreover, while a large increase in cadaverine content was observed in all three legume sprouts, the nutritionally beneficial polyamines (agmatine, spermidine, and spermine) were accumulated in microgreens, together with a lower cadaverine content. The authors also observed that daikon radish sprouts, in contrast to other ones, exhibited a nutritionally better profile of polyamines than the microgreens. Another interesting result of this study regards the enzymatic potential of fenugreek sprouts, since the authors found that homogenized fenugreek sprouts was effective in degrading exogenous putrescine, cadaverine, and tyramine at pH values above 5.

In the fifth paper, Manjula D. Ghoora and Nagarajan Srividya evaluated the “Effect of Packaging and Coating Technique on Postharvest Quality and Shelf Life of radish (*Raphanus sativus* L.) and roselle (*Hibiscus sabdariffa* L.) Microgreens” [14]. The authors studied the efficacy of two types of macro-perforated packaging (polyethylene terephthalate clamshell containers—PET-CS, and low-density polyethylene self-seal bags—LDPE-SSB) on the postharvest quality and shelf life of microgreens stored at 5 °C. Moreover, for the first time, spray- and dip-coating techniques were compared to study the effect of *Aloe vera* gel (AG) as an eco-friendly treatment on the postharvest quality and shelf life of radish and roselle microgreens. Physiological loss in weight, respiration rate, electrolyte leakage, color analysis, ascorbic acid content, microbial count, overall acceptability, and marketability were evaluated. Overall, the authors indicated that although macro-perforated PET-CS was found to be a comparatively better packaging than LDPE-SSB for postharvest quality maintenance during the storage of radish and roselle microgreens, LDPE-SSB could be used as an economical alternative in short distance markets and for sturdier microgreens. AG-coated microgreens had significantly lesser deteriorative postharvest changes and higher ascorbic acid content than the uncoated control, while AG spray coating maintained better overall acceptability and postharvest quality than AG dipping coating. Therefore, the authors concluded that AG spray coating could be suggested as an eco-friendly ergonomic pre-harvest treatment along with PET–CS for the enhancement of postharvest quality and shelf life in radish and roselle microgreens, with a high potential to be extended to other microgreens.

The sixth contribution is “Yield and Quality Characteristics of *Brassica* Microgreens as Affected by the NH_4_:NO_3_ Molar Ratio and Strength of the Nutrient Solution” by Onofrio Davide Palmitessa, Massimiliano Renna, Pasquale Crupi, Angelo Lovece, Filomena Corbo and Pietro Santamaria [15]. In this study, three *Brassica* genotypes (broccoli raab, broccoli and cauliflower) were fertigated using a nutrient solution with three different strength or with three different NH_4_:NO_3_ molar ratios percent (5:95, 15:85, and 25:75), starting from a Hoagland-like nutrient solution. Microgreen yields and content of inorganic ions, dietary fiber, proteins, α-tocopherol, and β-carotene were evaluated. The authors found that all three *Brassica* genotypes can be considered suitable for microgreen production, although micro cauliflower showed the highest yield, as well as a higher content of some mineral elements and α-tocopherol compared to other genotypes, while micro broccoli raab showed the fastest growth rate. Overall, the authors observed that the use of a Hoagland-like nutrient solution at half strength allowed them to obtain both high yield and desirable seedling height. On the other hand, the authors highlighted the possibility of producing microgreens of broccoli raab, broccoli and cauliflower by changing the NH_4_:NO_3_ molar ratio in the nutrient solution without negatively affecting yield, growing parameters and an important commercial characteristic such as the nitrate content, although the highest β-carotene content was found by using a nutrient solution with a NH_4_:NO_3_ molar ratio of 25:75.

In conclusion, the papers of this Special Issue cover a broad range of aspects and represent some of the recent research results regarding the topic of microgreens, which are gaining more and more interest not only for their nutritional value but also for their interesting organoleptic traits and commercial point of view. We think that this Special Issue may stimulate further research in this area.
